# Prognostic significance of L1 cell adhesion molecule in cancer patients: A systematic review and meta-analysis

**DOI:** 10.18632/oncotarget.13236

**Published:** 2016-11-09

**Authors:** Teng Hua, Shuangge Liu, Xiaoyan Xin, Zhishan Jin, Qibin Liu, Shuqi Chi, Xiaoxiao Wang, Hongbo Wang

**Affiliations:** ^1^ Department of Gynaecology and Obstetrics, Union Hospital, Tongji Medical College, Huazhong University of Science and Technology, Wuhan 430022, PR China; ^2^ Department of Surgery, Wuhan Pulmonary Hospital, Wuhan 430000, PR China

**Keywords:** cancer, L1 cell adhesion molecule, prognosis, meta-analysis

## Abstract

The L1 cell adhesion molecule (L1CAM) extensively participates in nervous system development and the malignant progression of human tumours. The prognostic value of L1CAM for the survival of patients with solid tumours remains controversial. The present meta-analysis was thus performed to highlight the relationship between L1CAM expression and prognosis in cancer patients. Relevant publications were identified after searching several widely used databases, including PubMed, EMBASE and the ISI Web of Science. A fixed-effect or random-effect meta-analytical model was employed to correlate L1CAM expression with different outcome measures in both entire tumours and stratified subgroups. 37 studies in total with 8552 patients were eligible for the final analysis. Combined hazard ratios (HRs) and 95% confidence intervals (CIs) suggested that high L1CAM expression had an unfavourable impact on overall survival (HR=2.06, 95%CI 1.65-2.57, P<0.001), disease-specific survival (HR=2.45, 95%CI 1.48-4.05, P<0.001), disease-free survival (HR=2.42, 95%CI 1.4-4.19, P=0.002) and progression-free survival/recurrence-free survival (HR=2.07, 95%CI 1.41-3.05, P<0.001). Subgroup analysis revealed a similar correlation in most tumour types. Overall, L1CAM might be an effective poor prognostic factor for patients with various tumour types.

## INTRODUCTION

Normally expressed in neural systems, L1 cell adhesion molecule (L1CAM) performs an essential role in nervous neural cell adhesion and migration, such as neurite outgrowth guidance, axon bundling, myelination, synaptogenesis and long-term potentiation [1]. Over the past decade, the knowledge of L1CAM in the cancer field has developed rapidly. The aberrant expression of L1CAM protein is closely correlated with the aggressive behaviour of several human malignancies. Mechanistic studies have indicated that forced changes in L1CAM expression distinctly alter cellular properties, including invasion, migration, proliferation and chemoresistance [2–4].

Although a majority of studies have shown that high L1CAM expression is interrelated with poor prognosis, the association between L1CAM overexpression and the outcome of cancer patients remains unknown. The overexpression of L1CAM in ovarian and endometrial cancer has a critical value in patient outcome prediction [5]. In addition, high L1CAM expression was associated with the progression of many other human cancers, including triple negative breast cancer [6], non-small lung cancer [7], pancreatic ductal adenocarcinoma [8], renal cell carcinoma [9], melanoma [10] and glioblastoma [11]. Conversely, Wachowiak and colleagues reported that the expression of L1CAM exhibited a favourable prognostic effect in children with neuroblastoma [12].

Although numerous studies have focused on the prognostic role of L1CAM expression in human solid tumours, most of these studies included only a limited number of patients, and the results of each individual study were not conclusive. We therefore performed a comprehensive meta-analysis to elucidate the prognostic value of L1CAM expression in all solid tumours using a relatively large sample size from 37 studies covering 8552 patients.

## RESULTS

### Study selection and characteristics

**Figure 1:**
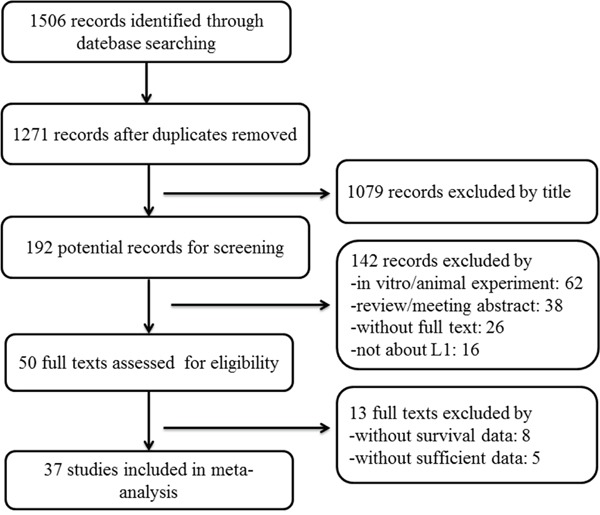
Flow chart of the literature search and selection

The detailed study selection is shown as Figure 1. A total of 1506 records were identified. Thirty-seven eligible studies [5–9, 12–43] encompassing 8552 patients were included in this meta-analysis. All studies were published between 2005 and 2016, and approximately 67.57% of them were published after 2010. The participants in the studies covered a wide variety of countries and cancer types. Most of the studies were from European (40.54%) and Asian (35.14%) countries, and the majority of the studies reported endometrial cancer (24.3%), followed by ovarian (10.8%) and colorectal cancer (10.8%); bile duct, hepatic, gallbladder, brain, lung, and vulvar cancers were only described once each. Immunohistochemical (IHC) staining was the predominant detection method for L1CAM, supplemented with RT-PCR and ELISA. For IHC, UJ127.11 and L1-14.10 were the most commonly used specific antibodies for L1CAM. In addition, >5% and >10% positive tumour cells and scores≥1 were all proper cut-off values for OS, and >10% positive tumour cells was a proper cut-off value for DFS, while no proper cut-off value was determined for PFS/RFS (Supplementary Table S1). The main characteristics of the 37 eligible studies are shown in Table 1. More specifically, most of the studies (27/37) described a positive relationship between high L1CAM expression and clinicopathological features, including advanced clinical stages, aggressive histologic grade, lymph node involvement and distant metastasis (data not shown). Additionally, 91.89% of the NOS scores for the included studies were ≥7, indicating a high quality for most of the studies. The detailed characteristics are listed in Supplementary Table S2.

**Table 1:** Main characteristics of studies exploring the relationship between L1CAM expression and tumor prognosis

Author	Year	Country	Cancer type	Stage/grade	No. of patients	Age Median(range)	Follow-up time Median(range)	Detection method	Cut-off	Outcomes
Allory	2005	France	Renal cell cancer	pT1-pT4	103	NA	34.7m(2-133)	IHC(mAb272)	>10 %	DFS
Kaifi	2006	Germany	GIST	NA	55	56.35y	41m	IHC(UJ127)	≥10%	RFS
Boo	2007	Korea	Colorectal cancer	I-IV	138	57.9y(18-82)	70.9m(3-129)	IHC(UJ127)	>5%	OS
Kaifi	2007	Germany	Colorectal cancer	pT1-pT4	247	65y	46m	IHC(UJ127)	score>1	DSS,OS
Wachowiak	2007	Germany	Neuroblastoma	Grade 1-3	66	30m	72m	IHC(UJ127)	NA	DFS,OS
Daponte	2008	Greece	Ovarian cancer	Grade 1-3	95	NA	3y	IHC(UJ127)	score>1	PFS
Zecchini	2008	Italy	Ovarian cancer	I-IV	211	NA	3.9y(0.14-11.47)	IHC(NA)	Membrane(+)	DFS,OS
KATO	2009	Japan	Colorectal cancer	I-IV	71	NA	34m(1-67)	IHC(UJ127)	score>2	OS
Kim	2009	Korea	Neuroendocrine tumor	I-IIIB	55	64y(24-80)	52m(2.6-133.7)	IHC(A10-A3)	>5%	DFS,OS
Kodera	2009	Japan	Gastric cancer	pT3	72	59.5y	6.11y(5-9.01)	IHC(UJ127)	≥10%	OS
Schroder	2009	Germany	Breast cancer	pT1-pT4	167	55.5y(29-85)	84m(8-169)	DNA-microarray	≥200	DFS,OS
Li	2009	Korea	cholangiocarcinoma	I-IV	75	65y(48-84)	16m(1-94)	IHC(A10-A3)	scores=+2/+3	PFS,OS
Ben	2010	China	PDAC	pT1-pT3	94	59y(31-79)	20m(3-45)	IHC(UJ127)	score≥30	OS
BERGMANN	2010	Germany	PDAC	pT3-pT4	110	63.2y(37-88)	20m(2-64)	IHC(14.10)	≥20%	OS
FANG	2010	China	Colorectal Cancer	Dukes A-D	142	55y(15-78)	>5y	IHC(UJ127)	>30%	OS
Huszar	2010	Germany	Endometrial cancer	IA-IIB	272	66.6y(32.7-87.7)	NA	IHC(14.10)	scores≥1	RFS
Tsutsumi	2011	Japan	PDAC	Grade 1-3	107	66y(37-80)	15.8m	IHC(UJ127)	≥10%	OS
Choi	2011	Korea	Gallbladder cancer	I-IV	69	67y(35-87)	37m(1-117)	IHC(A10-A3)	>5%	DFS,OS
Doberstein	2011	Germany	Renal cell cancer	pT1-pT3	282	63y(29-88)	40m(1-140)	IHC(14.10)	≥5%	OS
Tischler	2011	Switzerland	Non-small cell lung cancer	pT1-pT4	472	NA	25m(0-169,PFS); 51m(1-169,OS)	IHC(14.10)	scores≥1	PFS,OS
Zander	2011	Germany	GIST	NA	65	61y(28-81)	37m(0-273)	ELISA	>2 ng/ml	RFS
Bondong	2012	Germany	Ovarian cancer	IIA-IV	232	57y(18-85)	31m	ELISA	5.4ng/ul	PFS,OS
Guo	2012	China	Hepatocellular cancer	I-IV	130	NA	8.6y	IHC(UJ127)	scores≥4	DFS,OS
Chen	2013	China	Gastric cancer	I-IV	156	NA	30m(3-112)	IHC(5G3)	scores≥1	OS
ZHANG	2013	China	Breast cancer	Grade 1-3	97	53y(28-87)	51m(3-101)	IHC(14.10)	scores≥30	OS
Zeimet	2013	Austria	Endometrial cancer	IA-IB	1021	64y(34-96)	5.3y	IHC(14.10)	>10 %	DFS,OS
Bosse	2014	Netherlands	Endometrial cancer	IB-IIA	865	68.1y(41-90)	NA	IHC(14.10)	>10 %	OS
Doberstein	2014	Germany	Breast cancer	pT1-pT4	52	58.7y(33-84)	67.4m	IHC(14.10)	>10 %	DFS,OS
Ito	2014	Japan	Gastric cancer	I-IV	112	NA	NA	RT-PCR	NA	DFS,OS
Van	2016	Netherlands	Endometrial cancer	I-IV	116	66.3y(21-85)	28.6m(0.3-120)	IHC(14.10)	>10 %	RFS
Smogeli	2016	Norway	Endometrial cancer	IA-IB	388	NA	4.8y(0.1-8.8)	IHC(14.10)	>10 %	RFS,OS
Abdel	2016	Austria	Ovarian cancer	I-IV	138	62.8y	44m(1-242)	RT-PCR	>0.23	PFS,OS
Dellinger	2016	USA	Endometrial cancer	I-IV	545	64y(31-90)	23m(0-192)	RNA-seq	>5.37 fold	OS
Geels	2016	Netherlands	Endometrial cancer	I-IV	103	63y(24-86)	57m(0-148)	IHC(14.10)	>10 %	DSS,PFS
Notaro	2016	Austria	Endometrial cancer	I-IV	82	NA	11.6y(0.17-21.88)	RT-PCR	>10%	DFS,OS
Trietsch	2016	Netherlands	Vulvar cancer	I-IV	348	71y	4y	IHC(14.10)	≥5%	DSS,OS
Van	2016	Multiple	Endometrial cancer	I-IV	1199	64y(31-93)	62m(0-229)	IHC(14.10)	>10 %	DFS,OS

Abbreviations: GIST, gastrointestinal stromal tumors; PDAC, pancreatic ductal adenocarcinoma; NA, not available; IHC, immunohistochemistry; OS, overall survival; DFS, disease-free survival; DSS, disease-specific survival; RFS, recurrence-free survival; PFS, progression-free survival;

Cancer patients with excessive L1CAM expression had a poor prognosis. The association was significant and strong for all endpoints. The inverse correlation between L1CAM expression and outcome was dominant for DSS (HR=2.45, 95%CI 1.48-4.05, P=0.196) but weaker for OS (HR=2.06, 95%CI 1.65-2.57, P<0.001). As variations in cancer types can partly attributed to study heterogeneity, a subgroup analysis based on tumour type was performed. For most of the investigated cancer types, L1CAM demonstrated a significant prognostic value (Table 2).

**Table 2:** Results of subgroup meta-analysis of different tumor types reported

Tumor types	OS			DFS			PFS/RFS			DSS		
	No./case	HR(95%CI)	I^2^ (%)	No./case	HR(95%CI)	I^2^ (%)	No./case	HR(95%CI)	I^2^ (%)	No./case	HR(95%CI)	I^2^ (%)
Colorectal cancer	4/598	2.96(1.45-6.03)	59.9							1/247	2.64(1.49-4.66)	-
Neuroblastoma	1/66	1.49(0.92-2.40)	-	1/66	3.59(1.02-12.64)	-						
Ovarian cancer	3/581	1.25(1.07-1.46)	11.3	1/211	1.23(1.02-1.49)	-	3/465	1.85(1.34-2.56)	0			
Neuroendocrine tumor	1/55	6.11(1.73-21.66)	-	1/55	3.0(1.14-7.89)	-						
GIST	3/340	1.85(1.31-2.61)	0	1/112	0.93(0.45-1.93)	-	2/120	4.50(1.61-12.59)	0			
Breast cancer	3/316	2.23(0.94-5.30)	48.3	2/219	1.32(0.59-2.96)	18.9						
Cholangiocarcinoma	1/75	2.17(1.16-4.06)	-				1/75	1.38(0.64-3.0)	-			
PDAC	3/311	0.96(0.42-2.21)	85.5									
Gallbladder cancer	1/69	1.77(0.67-4.65)	-	1/69	3.50(1.15-10.69)	-						
Renal cell cancer	1/282	1.80(1.13-2.88)	-	2/103	1.33(0.19-9.09)	56.7						
NSCLC	1/472	1.31(1.01-1.70)	-				1/472	1.34(1.04-1.73)	-			
Hepatocellular cancer	1/130	3.27(1.29-8.30)	-	1/130	2.28(1.04-5.0)	-						
Endometrial cancer	6/4100	3.23(1.86-5.60)	86.2	3/2302	4.44(1.86-10.6)	88.6	4/879	3.93(0.90-17.26)	89.7	1/103	4.91(1.68-14.34)	-
Vulvar cancer	1/348	1.58(1.08-2.32)	-							1/348	1.7(0.97-2.97)	-
**Total**	30/7743	2.06(1.65-2.57)	81.8	13/3267	2.42(1.40-4.19)	89.5	11/2011	2.07(1.41-3.05)	73	3/698	2.45(1.48-4.05)	38.6

Overall HR (95%CI) was shown. No. /case refers to number of studies/patients included. Abbreviations: GIST, gastrointestinal Stromal Tumor; PDAC, pancreatic ductal adenocarcinoma; NSCLC, non-small cell lung cancer.

### L1CAM and overall survival

**Figure 2:**
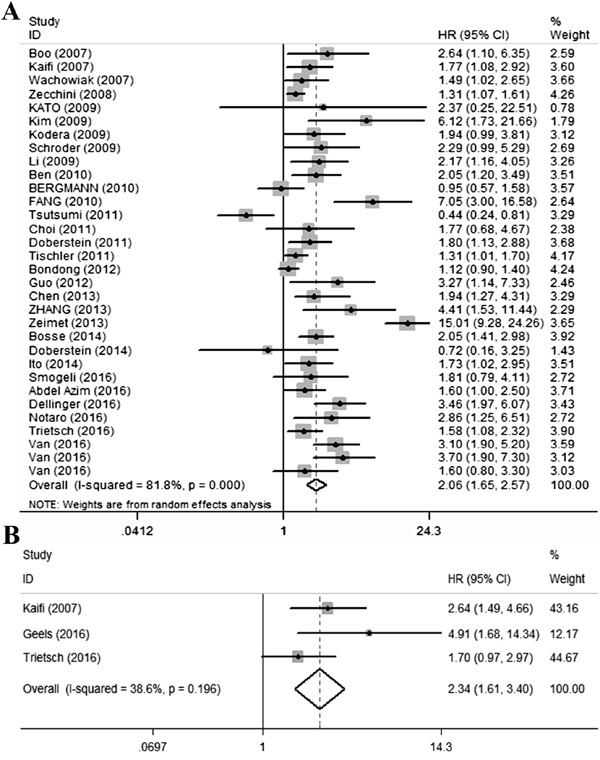
Qualitative meta-analysis of studies estimating the relationship between high L1CAM expression and the prognosis of patients with solid tumours **A.** Overall survival **B.** Disease-specific survival. Abbreviations: HR, hazard ratio; CI, confidence interval.

Thirty studies [5–8, 12, 14, 15, 17–25, 27, 28, 30–37, 39–41, 43] with data from 7743 patients were available to evaluate the effect of L1CAM expression on OS. The random-effects model was applied to calculate the pooled HR and 95%CI because of the significant heterogeneity among studies (I^2^=81.8%, P<0.01). Overall, high L1CAM expression was correlated with worse OS (HR=2.06, 95%CI 1.65-2.57, P<0.001) (Figure 2A). The subgroup analysis of different cancer types showed a similar significant association between L1CAM expression and OS in colorectal cancer, ovarian cancer, neuroendocrine tumours, gastric cancer, cholangiocarcinoma, renal cell cancer, non-small cell lung cancer, hepatocellular cancer, endometrial cancer and vulvar cancer. The remaining 4 cancer types, including neuroblastoma, breast cancer, pancreatic ductal adenocarcinoma and gallbladder cancer, also showed similar trends, although without statistical significance (Table 2).

### L1CAM and disease-specific survival

Only three studies [15, 38, 41] reported the DSS. A fixed-effects model was applied to calculate the pooled HR and 95%CI because low heterogeneity was detected (I^2^=38.6%, p=0.196). Patients with high L1CAM expression possessed a significantly shorter DSS than did those with low L1CAM expression (HR=2.34, 95%CI 1.61-3.4, P<0.001) (Figure 2B). Significant associations were also observed between colorectal cancer and endometrial cancer, but there was no significant association with vulvar cancer (Table 2).

### L1CAM and disease-free survival

**Figure 3:**
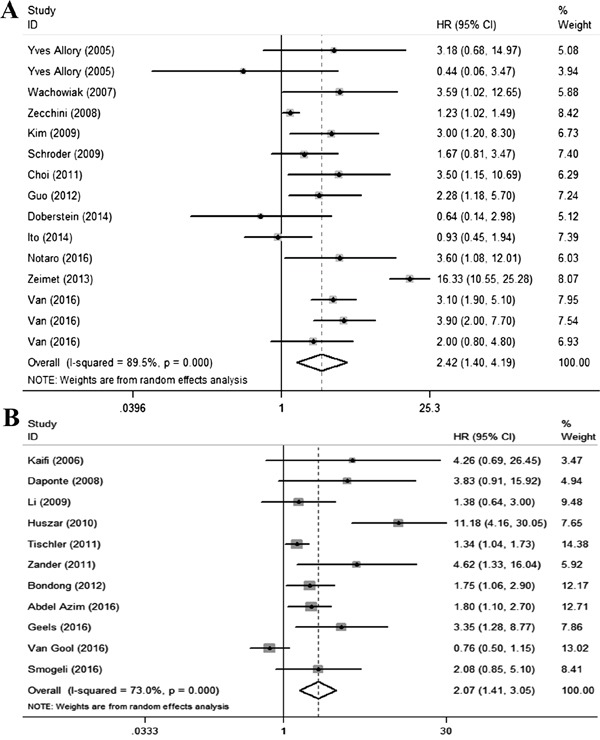
Qualitative meta-analysis of studies estimating the relationship between high L1CAM expression and the prognosis of patients with solid tumours **A.** Disease-free survival **B.** PFS/RFS. Abbreviations: PFS, progression-free survival; RFS, recurrence-free survival; HR, hazard ratio; CI, confidence interval.

DFS was reported in thirteen studies [6, 9, 12, 17, 19, 22, 27, 31, 33, 36, 39, 43] covering 3267 patients. Figure 3A shows the DFS outcome and demonstrates that cancer patients with high L1CAM expression have a shorter DFS than the control group (pooled HR=2.42, 95%CI 1.40-4.19, P=0.002). Subgroup analyses based on cancer types revealed significant reverse associations between L1CAM expression and DFS in neuroblastoma, ovarian cancer, neuroendocrine tumours, gallbladder cancer, hepatocellular cancer and endometrial cancer (Table 2).

### L1CAM and progression-free survival/recurrence-free survival

Due to the similar endpoints in progression-free survival and recurrence-free survival, a quantitative synthesis was performed in combination. A total of 11 studies [7, 13, 16, 21, 26, 29, 30, 37, 38, 40, 42] estimated the prognostic value of L1CAM expression on PFS/RFS. Consistent with the above results, significantly shorter PFS/RFS was observed in cancer patients expressing high levels of L1CAM (pooled HR=2.07, 95%CI 1.41-3.05, p<0.001) (Figure 3B). Subgroup analysis demonstrated similar effect of L1CAM expression on cancer types for PFS/RFS in ovarian cancer, gastric cancer, and NSCLC. However, the results were not consistent in extrahepatic cholangiocarcinoma and endometrial cancer (Table 2).

### Heterogeneity and sensitivity analysis

There was significant heterogeneity (I^2^>50%) between studies in OS, DFS and PFS/RFS analyses. A random-effect model was therefore adopted in these studies in addition to DSS. A meta-regression analysis with publication year, published country (from European or not), number of patients, and detected methods (IHC or not) as covariates was conducted. All covariates were preliminarily fit into the meta-regression model to identify covariates with highest p values; then, these variables were deleted one at a time to identify potential sources of heterogeneity. In terms of OS and PFS/RFS, none of these covariates were verified as a significant source of heterogeneity. Whereas the number of patients included in each individual study may be a source of heterogeneity for DFS (*Coef.*= 0.002, P=0.038) (Table 3). The sensitivity analysis indicated a stable result for both OS and PFS/RFS after using the leave-one-out method. However, the sensitivity analysis result was unstable for DFS after removing the study from Zeimet [33], which indicates that additional studies are needed to obtain more credible results (Supplementary Figure S1).

**Table 3:** Meta-regression results of L1CAM expression on patient outcomes

	OS			PFS/RFS			DFS		
	*Coef.*	*Std.Err*	*P*	*Coef.*	*Std.Err*	*P*	*Coef.*	*Std.Err*	*P*
**Pubication year**	0.053	0.057	0.365	-0.056	0.125	0.665	0.129	0.096	0.203
**Country**	-0.505	0.339	0.147	-0.123	0.842	0.887	-1.260	0.662	0.079
**NO. of patients**	0.001	0.001	0.105	0.000	0.002	0.896	0.002	0.001	0.038
**Detect method**	0.200	0.383	0.606	0.297	0.738	0.696	0.609	1.087	0.585

*Coef*, coefficient; *Std. Err*, standard error.

### Publication bias

**Figure 4:**
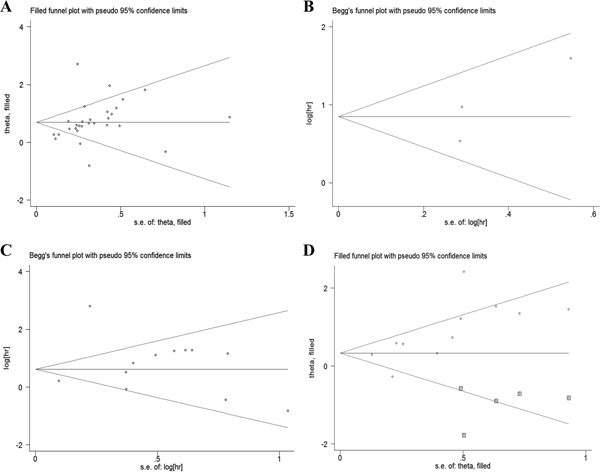
Begg's funnel plots for studies involved in the meta-analysis of L1CAM expression and the prognosis of patients with solid tumours **A.** Overall survival (Trim and Fill method) **B.** Disease-specific survival **C.** Disease-free survival **D.** PFS/RFS (Trim and Fill method, square dots display the filled studies). Abbreviations: PFS, progression-free survival; RFS, recurrence-free survival.

A funnel plot utilizing Egger's and Begg's rank correlation tests were conducted to evaluate the publication bias of the incorporated studies. The funnel plots for DSS and DFS (Figure 4B and 4C) were nearly symmetrical via visual inspection, and no significant publication bias was detected using Egger's test (P=0.390 for DSS, P= 0.471 for DFS), whereas both asymmetrical funnel plots and Egger's tests for OS and PFS/RFS indicated the existence of publication bias. To validate these results, a nonparametric Trim and Fill Method was employed. No “deleted studies” was filled for OS, and the estimated HR remained stable (Figure 4A). As for PFS/RFS, five studies were filled and no obvious asymmetry was observed in the funnel plot (Figure 4D), and the HR and 95%CI were not markedly altered. These results suggested that there were no significant publication bias between the eligible studies.

## DISCUSSION

Since the first record on the analysis of L1CAM expression in human cancer published in 2003 [44], more than sixty studies have explored the role of L1CAM expression in over eighteen types of tumours or malignancies in larger patient groups. The meta-analysis presented herein is the first comprehensive description of all reported studies investigating the impact of L1CAM expression in human tumours on prognosis. Overall, the results obtained for all of the endpoints evaluated showed that L1CAM might serve as an unexceptionable biomarker to predict the unfavourable outcomes for cancer patients. These results were further confirmed in a subgroup analysis stratified based on tumour types. More specifically, the prognostic value of L1CAM for patients with ovarian cancer, endometrial cancer, neuroendocrine tumours, colorectal cancer, and hepatocellular cancer were eminently remarkable. In addition, the predictive role of L1CAM in the prognoses of neuroblastoma, gallbladder cancer, renal cell cancer, gastrointestinal stromal tumours and several additional tumours is controversial, and some studies did not consistently show significance. Notably, few studies were available for the stratified analysis of several cancer types. Moreover, studies with larger sample sizes and high-quality data are warranted to validate these results.

The biological role of L1CAM may explain its poor prognostic value. *In vitro* or *in vivo* studies combined with the overexpression or depletion of L1CAM has shed light on the involvement of L1CAM in carcinogenesis and the development of several malignancies. The functional role of L1CAM in tumour cell invasion and motility primarily depends on ectodomain cleavage from membrane proximal proteolysis, binding partner alterations and integrin binding [2, 45]. Apart from the direct prognostic implications of L1CAM in cancer patients, L1CAM expression was positively associated with tumour progression, lymph node metastasis and the risk of loco regional or distant recurrence in most included cancer types. Recent studies have suggested a close connection between L1CAM and the epithelial-mesenchymal transition (EMT). High L1CAM expression was frequently observed at the invasive front of cancers with high vimentin and absent E-cadherin expression [26]. Both EMT and Wnt signalling regulators regulate L1CAM expression [46]. In addition, evidence has indicated a role for L1CAM in facilitating metastasis formation, pro-angiogenesis and resistance to chemotherapy [3, 4, 47, 48].

The present meta-analysis identifying a correlation between high L1CAM expression and worse outcome has some limitations. Despite the rigorous inclusion criteria, significant heterogeneity was detected in a majority of the meta-analyses with different endpoints. Using meta-regression tests, we identified sample size as a source of heterogeneity for DFS and eliminated publication year, ethnicity, sample size and detection method as heterogeneity sources for OS and PFS/RFS. There were also several other potential reasons for the observed heterogeneity. First, difficulties in obtaining a sufficient follow-up period and homogenous endpoints limited the accuracy of these results. Second, the distinct clinical behaviour, tumour staging, pathological grade and therapeutic regimen of the various solid tumours and one defined tumour type may have influenced the clinical outcomes because cancer patients with highly aggressive and advanced stage cancers are likely to have unfavourable prognoses. Third, the arbitrary cut-off points adopted in each of the included studies might have also served as potential sources of heterogeneity. Publication bias accounts for another important factor influence on the results. A comprehensive search and screening in different databases were conducted to minimize publication bias. Apart from the 37 identified eligible studies, five studies [44, 49–52] were excluded due to incomplete reporting. Thus, there was publication bias because not all of these studies were statistically significant. The present meta-analysis showed a publication bias for OS and PFS/RFS, according to the funnel plot and Egger's test. Nevertheless, the results remained stable after applying the trim and fill method; consequently, the effect of publication bias on this association might be minimal.

In conclusion, the present comprehensive meta-analysis of 37 studies with 8552 patients suggests that high L1CAM expression might be a prognostic factor for poor outcome in patients with various cancer types. This observation requires further multicentre prospective studies using a larger cohort sample size, adjusted individual data and a unified detection method to achieve a more persuasive conclusion.

## MATERIALS AND METHODS

### Literature searching strategies

The question of the meta-analysis was defined as: “what is the prognostic value of L1CAM expression in patients with tumours?” Accordingly, three distinctive keywords were identified, i.e., L1CAM, prognosis and tumour. The search algorithm was applied as the three keywords combined with a free text in any of the formulations or truncations. A comprehensive search was performed in PubMed (Supplementary Table S3), EMBASE and Web of Science databases prior to October 8, 2016.

### Screening of records

The first round of screening was conducted on the basis of title after duplicates removed, whereas further screening involved a detailed evaluation of the abstract and full-text. The following inclusion criteria were used: 1) papers investigating the role of L1CAM in the prognosis of human cancer; 2) a detailed protocol, including material source, methodology, quantification methods and threshold, was provided; 3) the full text was available and provided sufficient data for individual HR and 95% CI extracting or calculating; and 4) a minimum of 1 year of follow-up time for all endpoints. Studies presented with case reports, reviews, insufficient data, and the absence of statistical analysis were excluded.

### Data extraction and quality assessment

Two investigators independently extracted the data from each eligible paper. Several different parameters were collected, if provided, including the first author name, publication year, country, cancer type, cancer stage or grade, number of patients, median age of patients, median follow-up time, detection method, cut-off value, outcome definition, HR and 95% CI for the high L1CAM expression group versus the low L1CAM expression group. HR and 95% CI were estimated according to Tierney et al [53] when the univariate HR and 95%CI were unavailable. Multivariate HR and 95% CI were employed when both univariate and multivariate results were provided. The Newcastle-Ottawa-Scale (NOS) was adopted to assess the study quality of each individual study. The NOS score ranged from 0 to 9, and studies with NOS score ≥7 were defined as high-quality studies.

### Meta-analysis methods

Meta-analysis was performed using Stata version 12.0 (StataCorp, College Station, TX, USA). The following outcome endpoints were addressed: overall survival (OS), disease-free survival (DFS), disease-specific survival (DSS), progression-free survival (PFS), and recurrence-free survival (RFS). Pooled HR and 95%CI for each outcome endpoints were calculated. The fixed-effects model was adopted when no statistically significant heterogeneity was observed between studies (P_Q_>0.05, I^2^<50%), and when significant heterogeneity was observed across studies (P_Q_<0.05, I^2^>50%), the random-effects model was applied [54]. Meta-regression was conducted to identify the source when significant heterogeneity was observed. The assigned weight for each study was based on its inverse variance. The sensitivity analysis was performed using the leave-one-out method to explore the effects of each individual report on the pooled HR estimated. Further, Begg's funnel plot and Egger's test were conducted to identify publication bias [55]. Asymmetric funnel plots or P<0.05 in Egger's test suggest the existence of publication bias. The nonparametric trim and fill method [56] was used to validate the results when significant publication bias existed.

## SUPPLEMENTARY MATERIALS FIGURE AND TABLES


