# Supplementary material for: Prognostic significance of L1 cell adhesion molecule in cancer patients: A systematic review and meta-analysis
Source: Oncotarget. 2016 Nov 9;7(51):85196–207. doi: 10.18632/oncotarget.13236 (PMC5356729; doi:10.18632/oncotarget.13236)
Supplement: Supplementary file 1 [file oncotarget-07-85196-s001.pdf]

Prognostic significance of L1 cell adhesion molecule in cancer patients: A systematic review and meta-analysis

SUPPLEMENTARY FIGURE AND TABLES

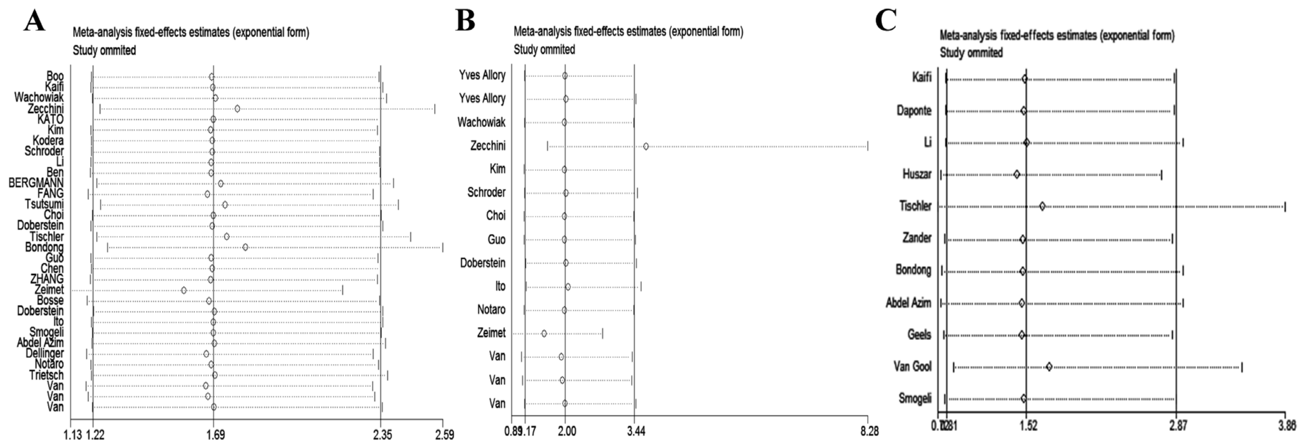

Supplementary Figure S1: Sensitivity analysis of L1CAM expression in the prognosis of patients with solid tumours. **A.** Overall survival **B.** Disease-free survival **C.** PFS/RFS. Abbreviations: PFS, progression-free survival; RFS, recurrence-free survival.

Supplementary Table S1: Subgroup meta-analysis exploring proper IHC cut-off value for patient with different outcome endpoints

Cut-off	OS		DFS		PFS/RFS	
	HR(95% CI)	P	HR(95% CI)	P	HR(95% CI)	P
>5%	1.902(1.398-2.587)	0.000				
>10%	2.221(1.201-4.109)	0.001	2.798(1.16-6.784)	0.022	1.862(0.757-4.576)	0.176
scores≥1	1.464(1.17-1.832)	0.011			3.651(0.822-16.21)	0.089

Quantitative analysis were performed when one cut-off value was adopted in more than 3 studies

Supplementary Table S2: Newcastle-Ottawa Scale to assess the quality of the included studies

study	Selection				Comparability	Outcome			
	Representative of cases	Selection of controls	Ascertainment of exposure	Outcomes present at start of study	Comparability the design or analysis	Assessment of outcome	Adequate follow-up time (≥ 1 year)	Adequacy of follow up (≥ 80%)	Total scores
Allory 2005	1	1	1	1	2	0	1	0	7
Kaifi 2006	1	1	1	0	2	1	1	1	8
Boo 2007	1	1	1	1	2	0	1	1	8
Kaifi 2007	1	1	1	0	2	1	1	0	7
Wachowiak 2007	1	1	1	0	2	1	1	0	7
Daponte 2008	1	1	1	0	2	0	1	0	6
Zecchini 2008	1	1	1	1	2	1	1	0	8
KATO 2009	1	1	1	1	2	0	1	0	7
Kim 2009	1	1	1	1	2	0	1	1	8
Kodera 2009	1	1	1	1	2	1	1	1	9
Schroder 2009	1	1	1	1	2	1	1	0	8
Li 2009	1	1	1	1	2	0	1	0	7
Ben 2010	1	1	1	1	2	1	1	0	8
BERGMANN 2010	1	1	1	1	2	1	1	0	8
FANG 2010	1	1	1	0	2	1	1	0	7
Huszar 2010	1	1	1	0	2	1	0	0	6
Tsutsumi 2011	1	1	1	0	2	1	1	1	8
Choi 2011	1	1	1	1	2	1	1	1	9
Doberstein 2011	1	1	1	1	2	1	1	0	8
Tischler 2011	1	1	1	0	2	1	1	0	7
Zander 2011	1	1	1	0	2	1	1	0	7
Bondong 2012	1	1	1	0	2	1	1	0	7
Guo 2012	1	1	1	0	2	1	1	0	7
Chen 2013	1	1	1	1	2	1	1	0	8
ZHANG 2013	1	1	1	1	2	1	1	0	8
Zeimet 2013	1	1	1	0	2	1	1	0	7
Bosse 2014	1	1	1	0	2	1	0	1	8
Doberstein 2014	1	1	1	0	2	1	1	0	8
Ito 2014	1	1	1	0	2	1	0	0	6
Van 2016	1	1	1	1	2	1	1	1	9
Smogeli 2016	1	1	1	1	2	1	1	0	8
Abdel 2016	1	1	1	1	2	1	1	0	8
Dellinger 2016	1	1	1	0	2	1	1	0	7
Geels 2016	1	1	1	0	2	1	1	0	7
Notaro 2016	1	1	1	1	2	1	1	0	8
Trietsch 2016	1	1	1	0	2	1	1	0	7
Van 2016	1	1	1	0	2	1	1	0	7

Supplementary Table S3: Literature search strategy to identify papers for the meta-analysis that describe an association between tumor L1CAM expression and prognosis

Keywords	Search algorithm	Results
**Tumor**	"Neoplasms"[Mesh] OR Neoplasm[Title/Abstract]) OR Tumors[Title/Abstract] OR Tumor[Title/Abstract]) OR Neoplasia[Title/Abstract] OR Cancer[Title/Abstract]) OR Cancers[Title/Abstract] OR Benign Neoplasms[Title/Abstract] OR Neoplasms, Benign[Title/Abstract] OR Benign Neoplasm[Title/Abstract] OR Neoplasm, Benign[Title/Abstract]	3393678
**L1CAM**	"Neural Cell Adhesion Molecule L1"[Mesh] OR NILE Glycoprotein[Title/Abstract] OR Glycoprotein, NILE[Title/Abstract] OR Nerve Growth Factor-Inducible Large External Glycoprotein[Title/Abstract] OR Nerve Growth Factor Inducible Large External Glycoprotein[Title/Abstract] OR CALL Protein[Title/Abstract] OR CamL1 Gene Product[Title/Abstract] OR Neural Adhesion Molecule L1[Title/Abstract] OR L1 Cell Adhesion Molecule[Title/Abstract] OR L1CAM[Title/Abstract] OR NILE Protein[Title/Abstract] OR Cell Adhesion Molecule L1[Title/Abstract] OR Cell Surface Glycoprotein L1[Title/Abstract] OR NGF-Inducible Glycoprotein[Title/Abstract] OR Glycoprotein, NGF-Inducible[Title/Abstract] OR NGF Inducible Glycoprotein[Title/Abstract] OR F11 Glycoprotein[Title/Abstract]	2021
**Prognosis**	Prognosis OR outcome OR survival	3025068
**Tumor AND L1CAM AND Prognosis**		157
